# Deep brain stimulation for Parkinson’s disease–related postural abnormalities: a systematic review and meta-analysis

**DOI:** 10.1007/s10143-022-01830-3

**Published:** 2022-07-05

**Authors:** Philipp Spindler, Yasmin Alzoobi, Andrea A. Kühn, Katharina Faust, Gerd-Helge Schneider, Peter Vajkoczy

**Affiliations:** 1 Department of Neurosurgery, Charité University Medicine Berlin, Charitéplatz 1, 10117 Berlin, Germany; 2 Department of Neurology, Charité University Medicine Berlin, Berlin, Germany

**Keywords:** Deep brain stimulation, Parkinson’s disease, Posture, Spine, Neurosurgery

## Abstract

Deep brain stimulation (DBS) has become a well-established treatment modality for Parkinson’s disease (PD), especially regarding motor fluctuations, dyskinesias, and tremor. Although postural abnormalities (i.e., Camptocormia [CC] and Pisa syndrome [Pisa]) are known to be a major symptom of PD as well, the influence of DBS on postural abnormalities is unclear. The objective of this study is to analyze the existing literature regarding DBS for PD-associated postural abnormalities in a systematic review and meta-analysis. In compliance with the Preferred Reporting Items for Systematic Reviews and Meta-Analyses (PRISMA) guidelines, we conducted a systematic review and meta-analysis of 18 studies that reported the effect of DBS regarding postural abnormalities. After screening of 53 studies, a total of 98 patients (44 female, 53 males, 1 not reported; mean age: 62.3, range 30–83 years) with postural abnormalities (CC *n* = 98; Pisa *n* = 11) were analyzed from 18 included studies. Of those patients, 94.9% underwent STN-DBS and 5.1% had GPi as DBS target area. A positive outcome was reported for 67.8% with CC and 72.2% with Pisa. In the meta-analysis, younger age and lower pre-operative UPDRS-III (ON/OFF) were found as positive predictive factors for a positive effect of DBS. DBS might be a potentially effective treatment option for PD-associated postural abnormalities. However, the level of evidence is rather low, and definition of postoperative outcome is heterogenous between studies. Therefore larger, prospective trials are necessary to give a clear recommendation.

## Introduction

Parkinson’s disease (PD) affects 1% of the population over the age of 60, with increasing prevalence, and is therefore the second most common neurodegenerative disease worldwide [1]. Beside characteristic features of PD (bradykinesia, rigidity, and tremor), one of the most noticeable signs of PD patients is abnormality in their posture, with functional alterations of the spine [2]. The clinical phenotype of postural abnormalities is variable: while Camptocormia (CC) presents in the sagittal, Pisa syndrome (Pisa) is observed in the coronal plane [3]. CC is defined by an abnormal thoraco-lumbar spinal flexion which presents while standing and walking and is alleviated in a recumbent position. Most authors define CC by an arbitrary angle of at least 30–45° flexion of the thoraco-lumbar spine in the standing position [4, 5]. Pisa is defined as a reversible lateral deviation of the spine (> 10°) with a corresponding tendency to lean to one side. It is not to be confused with scoliosis, in which lateral bending is caused by an S-shaped curvature and rotation of the spine. Like CC, Pisa occurs when standing and disappears in the recumbent position [6, 7]. It is obvious that these postural abnormalities subsequently lead to back pain and degenerative alterations of the spine. With the increasing prevalence of PD and the associated degenerative spinal conditions, the demand for spinal surgery in patients with PD increases rapidly. However, the results of spinal surgery for degenerative spinal conditions in patients with PD are disappointing with failure rates of 25.8–100% [8–12]. As an alternative treatment strategy for the underlying pathomechanism, some authors have described the influence of deep brain stimulation on PD-related postural abnormalities [13–30]. We aim to present a systematical review of the current literature. Moreover, we conducted a meta-analysis to evaluate predictive factors for a successful outcome of DBS.

## Methods

### Search strategy

To collect fundamental data, the systematic review was done in accordance with the criteria outlined in the Preferred Reporting Items for Systematic Reviews and Meta-Analyses (PRISMA) 2009 guidelines [31]. Institutional Review Board approval and/or patient consent were not required. Two reviewers (PS and YA) conducted a computerized search between January 2002 and January 2022 on the PubMed and Web of Science databases. The following algorithm was developed as search strategy: (deep brain stimulation [Title/Abstract]) AND (spine [Title/Abstract]) AND (Parkinson [Title/Abstract]), Filters: Humans, English for the PubMed search and ((TS = (deep brain stimulation)) AND TS = (spine)) AND TS = (parkinson) for the Web of Science search. The reference lists of the included studies were searched to obtain additional articles.

### Study selection

Studies were included if they met the following criteria: (1) human patients, (2) English language, (3) peer-reviewed original articles with full-text available (reviews, systematic reviews, and meta-analyses were excluded), (4) reported postural abnormalities (i.e., PS and CC), (5) performance of Deep Brain Stimulation. Studies that did not meet all inclusion criteria were excluded.

### Data extraction

The following study characteristics were extracted from each included article: (1) author and year of publication, (2) study design, (3) number of patients, (4) sex of patients, (5) duration of follow-up, (6) duration between diagnosis of PD and DBS, (7) duration between onset of postural abnormity and DBS, (8) type of postural abnormity, (i.e., CC or Pisa), (9) target structure of DBS, (10) type of outcome parameter, (11) UPDRS-III pre- and post-OP.

To avoid extraction errors, two reviewers (PS and YA) independently extracted data from the eligible articles. Any discrepancies were discussed and resolved with a third reviewer (PV).

### Statistical analysis

According to the reported primary outcomes of the included studies, a relative reduction of > 50% of the thoraco-lumbar angle (TLA) respectively and an absolute value of < 30° of the TLA post-DBS were defined as positive outcome (Table 1). Those parameters were dichotomized (i.e., > 50% reduction vs. < 50% reduction and < 30° vs > 30°) and stratified by each of the assessed variables via Student’s *t*-test (with a significance level of *p* < 0.05) to identify possible associations with outcome (Table 2). For the meta-analysis, the continuous variables which demonstrate a significant difference for the outcome parameters were subjected. Heterogeneity of study outcomes between the included studies was calculated by Cochran’s *Q* and *I*^2^ statistics and consequently it was defined whether fixed or random effects models were appropriate. Inverse variance tests calculated mean difference and confidence intervals of 95%. The results were visualized by forest plot asymmetry.

**Table 1:** Summary of studies that reported DBS as treatment for Parkinson’s disease–related postural abnormalities

Author, year	Type of study	Level of evidence	PD patients with postural abnormity	Intervention	Outcome parameter	Result-No. of patients	Length of follow-up
Anderson et al., 2019	Case report	4	1 (Pisa)	GPi-DBS	Cobb-angle on X-ray	1/1 effective	4 years
Asahi et al., 2011	Case series	4	4 (CC)	STN-DBS	TLA on photographs	3/4 effective	25.8 (18–40) months
Azher et al., 20,015	Case report	1	1 (CC)	STN-DBS	TLA on photographs	1/1 non- effective	N/A
Capelle et al., 2011	Case series	4	3 (CC)	2 STN-DBS 1 GPi-DBS	VAS, BFM-RS motor-sub score	2/3 effective	21.3 (12–36) months
Ekmekci et al., 2016	Case report	4	1 (CC)	STN-DBS	S-LANNS pain scale, BFM-RS, TLA on photographs	1/1 effective	1 year
Hellmann et al., 2016	Case report	4	1 (CC)	STN-DBS	Ability to stand and walk (physical examination)	1/1 effective	10 months
Lyons et al., 2012	Case report	4	1 (CC)	STN-DBS	Report about back pain, TLA on photographs	1/1 effective	5 years
Micheli et al., 2005	Case report	4	1 (CC)	GPi-DBS	TLA on photographs	1/1 effective	14 months
Sakai et al., 2017	Retrosp obs cohort	3	14 (CC)	STN-DBS	TLA on photographs	4/14 effective 5/14 part effective 5/14 non-effective	6 months
Sako et al., 2009	Case series	4	6 (CC)	STN-DBS	TLA on photographs	6/6 effective	16.8 (5–46) months
Schaebitz et al., 2003	Case report	4	1 (CC)	STN-DBS	TLA on photographs	1/1 effective	N/A
Schulz-Schaeffer et al., 2015	Retrosp obs cohort	3	25 (CC)	STN-DBS	VAS, TLA on photographs	13/25 effective 12/25 non-effective	N/A
Thani et al., 2011	Case report	4	1 (CC)	GPi-DBS	sSHK-angle and sHSH-angle on photographs	1/1 effective	14 months
Umemura et al., 2010	Case series	18	8 (CC) 10 (Pisa)	STN-DBS	UPDRS-III item 28	CC: 4/8 effective 2/8 part effective 2/8 non-effective Pisa: 6/10 effective 1/10 part-effective 3/10 non-effective	12 months
Upadhyaya et al., 2010	Review incl. case reports	4	2 (CC)	1 STN-DBS 1GPi-DBS	Anecdotal	2/2 non-effective	19.5 (14–24) months
Yamada et al., 2006	Case report	4	1 (CC)	STN-DBS	TLA on photographs	1/1 effective	20 months
Yamada et al., 2016	Prosp trial	2	17 (CC)	STN-DBS	S-E activity scale, TLA on photographs	4/17 effective 8/17 part effective 5/17 non-effective	36,5 (13–67) months
Lai et al., 2021	Retrosp obs cohort	3	11 (CC)	GPi-DBS	TLA and UCA on photographs	Effective by 40.4% (TLA) and 22.8% (UCA)	7.3 (± 3.3) months

*PD* Parkinson’s disease, *CC* Camptocormia, *Pisa* Pisa syndrome, *GPi* globus pallidus internus, *STN* subthalamic nucleus, *TLA* thoraco-lumbar angle, *VAS* Visual Analogue Scale, *BFM-RS* Burke-Fahn-Mardsen Rating Scale, *sSHK angle* sagittal shoulder-hip-knee angle, *sHSH* sagittal shoulder-hip-knee angle, *UPDRS-III* Unified Parkinson’s Disease Rating Scale Part III, *S-E* Schwab-England, *UCA* Upper Camptocormia Angle

**Table 2:** Summary of preliminary data results

	Absolute TLA post STN-DBS			Relative TLA reduction post STN-DBS		
Parameter	< 30° = impr	> 30° = no impr	p-value	> 50% reduction = impr.	< 50% reduction = no impr	p-value
Age, mean ± SD	59.7 ± 6.6	67.1 ± 5.8	< 0.0005	61.4 ± 7.6	66.2 ± 5.9	< 0.05
Sex			0.4			0.6
Female	8	16		11	13	
Male	9	10		7	12	
Duration of PD, mean ± SD (yrs.)	12.1 ± 4.9	12.5 ± 5.1	0.8	11.5 ± 4.9	13.0 ± 5.0	0.4
Duration of CC, mean ± SD (yrs.)	3.1 ± 2.0	4.4 ± 2.4	0.1	2.9 ± 1.6	4.5 ± 2.6	0.1
UPDRS-III ON, pre-OP	27.4 ± 11.3	27.8 ± 12.1	0.9	31 ± 13.8	23.5 ± 5.5	0.1
UPDRS-III OFF, pre-OP	44.7 ± 21.9	42.5 ± 10.7	0.8	48.4 ± 20.8	38.3 ± 10.2	0.2
UPDRS-III ON, post-OP	14.1 ± 8.0	25.8 ± 12.3	< 0.01	18.1 ± 13.9	20.7 ± 6.6	0.6
UPDRS-III OFF, post-OP	23.8 ± 14.4	36.5 ± 14.9	< 0.05	26.1 ± 15.4	33.4 ± 15.1	0.3
LED (mg), pre-OP mean ± SD	744.9 ± 334.3	577.7 ± 301.3	0.3	696.1 ± 416.6	624.2 ± 230.7	0.6
LED (mg), post-OP mean ± SD	301.8 ± 111.9	309.5 ± 142.6	0.9	265.4 ± 98.3	329.4 ± 132.4	0.3

According to the included studies, change of the TLA was defined as outcome parameters in two ways: (a) absolute TLA < 30° vs. > 30° and (b) relative change of TLA > 50% vs < 50%. *TLA* thoraco-lumbar angle, *PD* Parkinson’s disease, *CC* Camptocormia, *UPDRS-III* Unified Parkinson’s Disease Rating Scale, *LED* levodopa equivalent dose. Parameters with statistical significance (*p* < 0.05) were chosen for subsequent meta-analysis

Unpaired, two-tailored Student’s *t*-tests (with a significance level of *p* < 0.05) were performed with the use of GraphPad Prism version 8.4.2 for Mac, GraphPad Software, San Diego, CA, USA, www.graphpad.com. Meta-analysis was performed with the use of Review Manager (RevMan) [for Mac] version 5.4, The Cochrane Collaboration, 2020.

## Results

### Search results and study selection

**Fig. 1:**
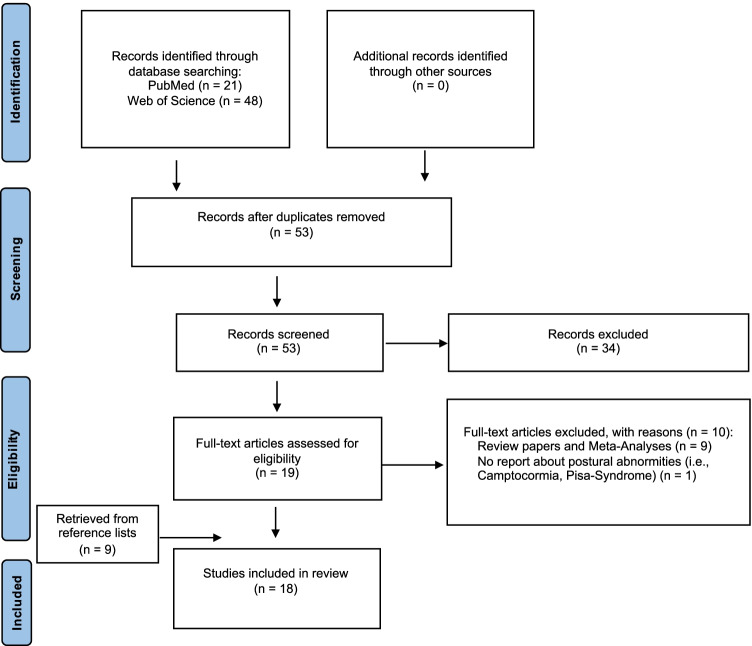
PRISM flowchart of the study selection process

After removal of the duplicates from the initial database, 53 articles underwent the first screening process of titles and abstracts. Subsequently, 19 potentially relevant articles were identified that underwent full-text review and screening against the inclusion criteria. Ten articles were excluded (review articles and meta-analyses *n* = 9; no report about postural abnormalities *n* = 1), resulting in a total of nine articles. From the reference list, nine additional articles which were not found in the initial algorithm-based search were included, resulting in 18 articles that were finally chosen to be included in the analysis. A RRISM flowchart of the study selection process is represented in Fig. 1.

### Study characteristics

The included studies have been conducted between 2003 and 2021. Regarding study designs, nine case reports [5, 13, 17–20, 23, 25, 28] (i.e., 50%), four case series [14, 16, 22, 26] (i.e., 22.2%), three retrospective observational cohort studies [21, 24, 30] (i.e., 16.6%), one review article including two reported cases [27] (i.e., 5.6%), and one prospective trial [29] (i.e., 5.6%) were assessed. Fifteen studies [13, 14, 16–21, 25, 27–30, 32] reported follow-up data with a mean follow-up time of 21.5 months (range 5–67 months). A mean of 6.1 (range 1–25) patients per study was detected. The study characteristics are presented in Table 1.

### Outcome parameter

There were several primary outcome parameters to define improvement, partial improvement, or no improvement through DBS among the included studies. (1) Five studies reported clinical outcomes by standardized scores (i.e., Visual Analogue Scale [VAS] [16, 24], Burke-Fahn-Mardsen Dystonia Rating Scale |BFM-RS] [16, 17], S-LANNS Score [17], Schwab-England activity of daily living scale [S-E] [29], and special consideration of Unified Disease Parkinson Rating Scale [UPDRS]-III item 28 score [26]), four studies anecdotally reported the clinical outcome without standardizes scores [13, 18, 19, 27], and nine studies did not take the clinical course into account. (2) Changes of the sagittal thoraco-lumbar angle (TLA) (or shoulder-hip knee and head-shoulder-hip angle) assessed by photographs were assessed in 13 (i.e., 72,2%) studies [5, 14, 17, 19–25, 28–30], with outcome parameter either defined as improvement in case of > 50% relative reduction of the TLA or as absolute value < 30° of the TLA at last follow-up (Table 1). Two studies further distinguished improvement as effective and partially effective: in Sakai et al., partially effective was defined when the TLA became < 30° after DBS but did not last for > 6 months [21]. Yamada et al. defined partially effective when the TLA improved > 20° but < 50° [29].

### Patient demographics and outcome

**Fig. 2:**
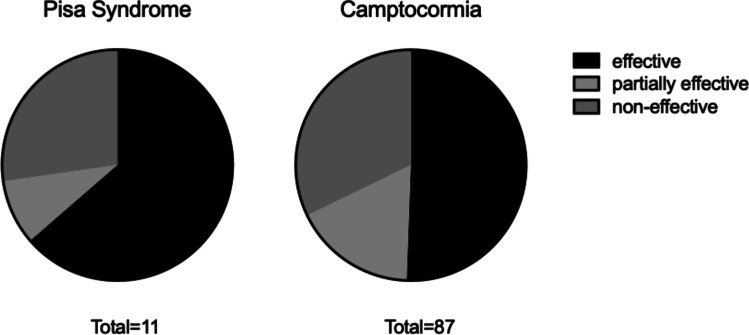
Proportions of effective, partially effective, or non-effective outcome after DBS with respect to postural abnormalities (i.e., Pisa syndrome and Camptocormia)

A total of 98 patients (44 female, 53 males, 1 not reported; mean age: 62.3, range 30–83 years) with postural abnormalities (*n* = 98 with CC and *n* = 11 with Pisa) were included in the analysis. Of those 93 patients (i.e., 94.9%) who underwent STN-DBS, in 5 patients (i.e., 5.1%), the GPi was chosen as DBS target area. Depending on what was defined as outcome parameter in each study, DBS was effective in seven (i.e., 63.6%) patients, partially effective in one (i.e., 9.1%) patient, and non-effective in three (i.e., 27.3%) patients with Pisa. For patients with CC, DBS was effective in 44 (i.e., 50.6%) patients, partially effective in 15 (i.e., 17.2%) patients, and non-effective in 28 (i.e., 32.2%) patients (Fig. 2, Table 3). Excluding case reports from the analysis resulted in 60% effective, 10% partially effective, and 30% non-effective regarding PS and 48.1% effective, 19% partially effective, and 32.9% non-effective for CC.

**Table 3:** Individual patient characteristics from the included studies

Author, year	Age/sex	DBS target	Outcome	Follow-up period
Pisa syndrome				
Anderson, 2019	73/M	GPi	Effective	4 years
Umemura, 2019	71/F	STN	Effective	12 months
	75/F	STN	Effective	12 months
	60/F	STN	Effective	12 months
	69/M	STN	Effective	12 months
	56/F	STN	Effective	12 months
	59/F	STN	Effective	12 months
	61/F	STN	(Partially) Effective	12 months
	73/F	STN	Non-effective	12 months
	71/M	STN	Non-effective	12 months
	58/F	STN	Non-effective	12 months
Camptocormia				
Asahi, 2011	60/M	STN	Effective	18 months
	69/M	STN	Effective	21 months
	61/F	STN	Effective	40 months
	61/F	STN	Non-effective	24 months
Azher, 2005	N/A	STN	Non-effective	N/A
Capelle, 2011	73/M	STN	Effective	16 months
	65/M	STN	Effective	12 months
	64/M	GPi	Effective	36 months
Ekmekci, 2016	51/F	STN	Effective	12 months
Hellmann, 2006	53/M	STN	Effective	10 months
Lyons, 2012	63/F	STN	Effective	5 years
Micheli, 2005	72/M	GPi	Effective	14 months
Sakai, 2017	56/M	STN	Effective	6 months
	71/F	STN	Effective	6 months
	71/M	STN	Effective	6 months
	49/M	STN	Effective	6 months
	70/M	STN	(Partially) Effective	6 months
	70/F	STN	(Partially) Effective	6 months
	61/F	STN	(Partially) Effective	6 months
	59/M	STN	(Partially) Effective	6 months
	61/M	STN	(Partially) Effective	6 months
	60/M	STN	Non-effective	6 months
	69/M	STN	Non-effective	6 months
	65/F	STN	Non-effective	6 months
	73/F	STN	Non-effective	6 months
	74/F	STN	Non-effective	6 months
Sako, 2009	71/F	STN	Effective	46 months
	64/M	STN	Effective	15 months
	55/F	STN	Effective	18 months
	53/F	STN	Effective	5 months
	65/M	STN	Effective	8 months
	53/F	STN	Effective	9 months
Schaebitz, 2003	65/M	STN	Non-effective	N/A
Schulz-Schaeffer, 2015	49.8/11xM,2xF	STN	13 effectives	N/A
	50.8/10xM,2xF	STN	12 non-effectives	N/A
Thani, 2011	57/F	GPi	Effective	14 months
Umemura, 2010	63/F	STN	Effective	12 months
	60/F	STN	Effective	12 months
	59/M	STN	Effective	12 months
	63/F	STN	Effective	12 months
	63/F	STN	(Partially) Effective	12 months
	66/F	STN	(Partially) Effective	12 months
	68/F	STN	Non-effective	12 months
Upadhyaya, 2010	59/M	STN	Non-effective	24 months
	59/M	GPi	Non-effective	15 months
Yamada, 2006	71/F	STN	Effective	20 months
Yamada, 2016	73/F	STN	Effective	22 months
	54/M	STN	Effective	19 months
	64/M	STN	Effective	28 months
	64/F	STN	Effective	34 months
	58/M	STN	(Partially) Effective	53 months
	72/M	STN	(Partially) Effective	52 months
	74/F	STN	(Partially) Effective	27 months
	56/F	STN	(Partially) Effective	57 months
	72/F	STN	(Partially) Effective	36 months
	66/M	STN	(Partially) Effective	36 months
	59/F	STN	(Partially) Effective	24 months
	64/F	STN	(Partially) Effective	18 months
	73/F	STN	Non-effective	67 months
	67/M	STN	Non-effective	58 months
	69/F	STN	Non-effective	60 months
	77/F	STN	Non-effective	13 months
	66/M	STN	Non-effective	17 months

*M* male, *F* female, *GPi* globus pallidus internus, *STN* subthalamic nucleus

### Meta-analysis results

**Fig. 3:**
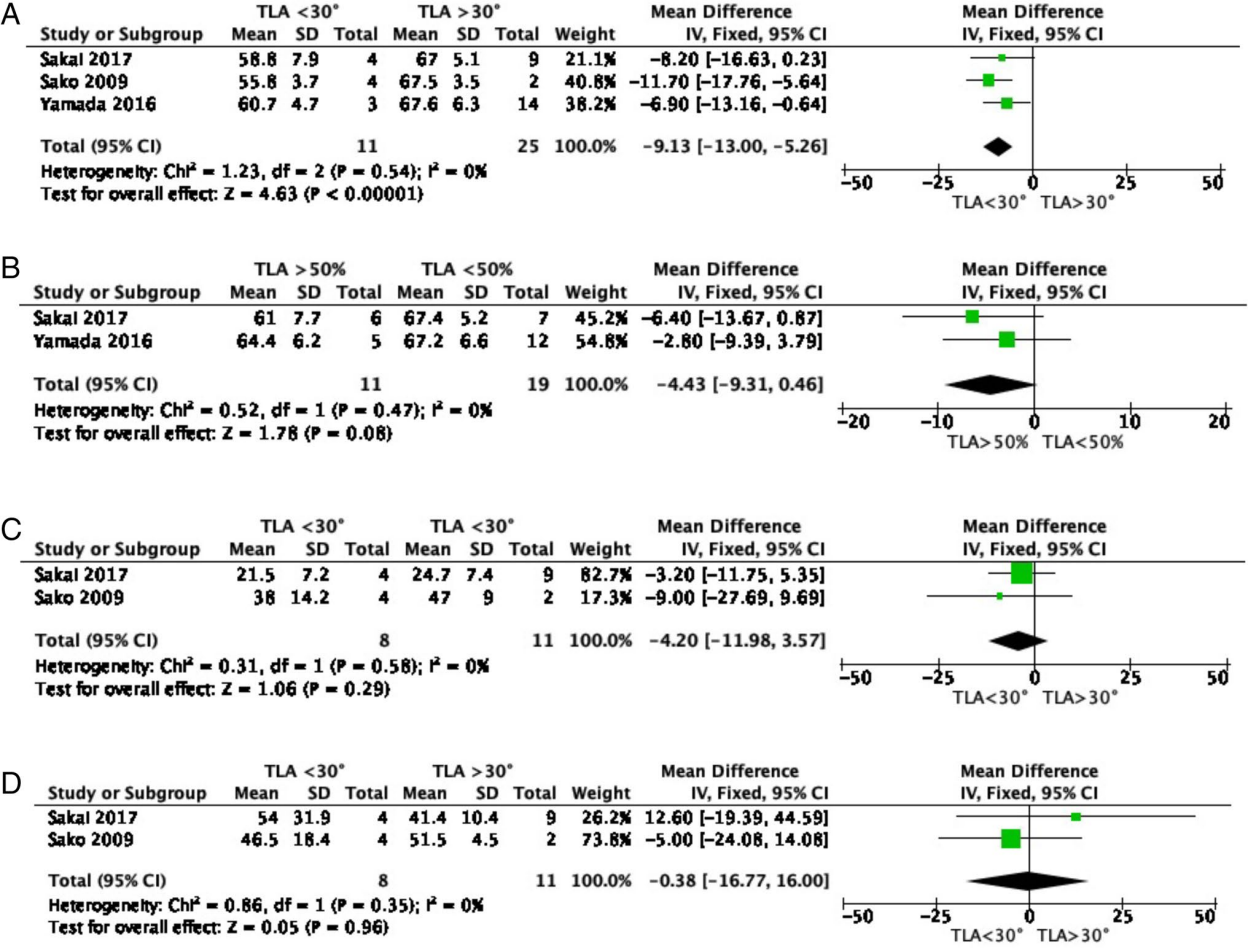
Meta-analysis of predictive parameters for positive outcome (i.e., absolute TLA < 30° or relative TLA improvement > 50%). **A** Mean (± SD) of patient age with respect to absolute TLA after DBS. **B** Mean (± SD) of patient age with respect to relative TLA reduction after DBS. **C** Pre-OP UPDRS (ON) with respect to absolute TLA after DBS. **D** Pre-OP UPDRS (OFF) with respect to absolute TLA after DBS

Three studies were included in the meta-analysis [21, 22, 29] and 15 studies were excluded due to low number of participants and/or missing demographic parameters. The preliminary analysis was analyzed according to the outcome parameters of the studies (improvement through DBS defined as absolute TLA < 30° or amelioration of the TLA > 50% post-DBS). Younger age was found as positive predictor for beneficial effect of DBS on both outcome parameters (mean age 59.7 ± 6.6 vs 67.1 ± 5.8 years, *p* < 0.0005 for absolute TLA < 30° and 61.4 ± 7.6 vs 66.2 ± 5.9 years, *p* < 0.05 for relative TLA reduction > 50%) (Table 2). Inverse variance analysis revealed a difference of 9.1 (95% CI 5.3–13.0) years between patients with absolute TLA < 30° vs. TLA > 30° and of 4.4 (95% CI 0.5–9.3) years between patients with > 50% vs. < 50% relative TLA improvement (Fig. 3A and B). Lower pre-operative UPDRS-III (ON/OFF) was found as another positive predictive factor (14.1 ± 8.0 vs. 25.8 ± 12.3, *p* < 0.01 for UPDRS-III ON and 23.8 ± 14.4 vs. 36.5 ± 14.9, *p* < 0.05 for UPDRS-III OFF) in case of absolute TLA improvement (Table 2). In the inverse variance analysis, the mean difference of UPDRS-III ON/OFF between patients with absolute TLA < 30° vs TLA > 30° was 4.2 (95% CI − 12.0 to 3.6) and 0.4 (95% CI − 16.8 and 16.0) (Fig. 3C and D).

## Discussion

After 53 articles have been screened, 18 were included in the analysis, of those three underwent subsequent meta-analysis. The following main findings were detected: (1) Postural abnormalities associated with PD improved, at least partially, in 67.8% (CC) and 72.2% (Pisa) of patients following DBS. (2) Younger age was found as a positive predictive factor for a beneficial effect of DBS. (3) Lower pre-OP UPDRS-III (ON and OFF) was associated with better outcome following DBS.

DBS has become a well-established treatment option for PD over the past decades. Especially patients with motor fluctuations, dyskinesias secondary to chronic levodopa and those with refractory and marked tremor benefit from DBS [33]. Although postural abnormalities are known to be a major symptom of PD [2], which in several cases give rise to spinal deformities [34], there is a gap of knowledge regarding the influence of DBS on postural abnormalities associated with PD. We aimed to summarize the existing literature by performing a systematic review. We found very heterogeneous approaches to describe the influence of DBS on postural deformities: The definition of appropriate outcome parameter varied between the included studies. While some studies examined clinical parameters, other studies used photographs to evaluate the patients’ TLA prior and after DBS. Even within this rough subdivision of outcome parameters, there were significant differences. Clinical parameters were rarely expressed by standardized scores, rather than by anecdotal reports, which precludes subjective comparisons. Regarding TLA, which was used as outcome parameter in 72.2% of the studies, some authors defined a positive effect of DBS by a relative improvement of the TLA > 50% [24, 29], other authors describe a post-OP absolute TLA < 30° as positive effect [14, 21], and the remaining authors reported a positive effect or non-effect, but without defining this precisely.

Three studies reported aspects of the paraspinal muscles associated with postural abnormalities: Asahi et al. described a higher density of paraspinal muscle (measured by CT scans) in patients that improved through DBS [14]. This finding was confirmed by Sakai et al., who performed MRI scans of the lumbar spine and detected a larger cross-sectional area of paraspinal muscle in patients with positive effect of DBS [21]. Schaebitz et al. found myopathy confined to the paraspinal muscles in a small case series of PD patients with CC [23].

Schulz-Schaffer et al. and Yamada et al. found that a longer duration of CC prior to DBS was associated with less improvement of the TLA [24, 29]. This correlation was further analyzed in a meta-analysis by Chan et al.[35] who described a duration of CC < 2 years predictive for better outcomes. We did not confirm those results, since Schulz-Schaffer et al. did not reveal individual patient characteristics and therefore were not eligible four our meta-analysis.

### Limitations

There are several limitations to this study. First, the evidence is mostly limited to case series and reports. To avoid reporting artificially too positive results from case reports, we performed an analysis in which patients from case reports were excluded. However, this did not fundamentally change the generally rather positive overall result. Moreover, there are three retrospective observational cohort studies and one prospective trial, yet neither of those studies is designed to show superiority of DBS over conservative treatment. Second, the heterogenous definition of outcome parameter impedes the comparison of the studies with one another. An objective comparison can only be made if the individual parameters of the patient are given.

## Conclusion

We systematically reviewed the existing literature regarding the effect of DBS on PD-associated postural abnormalities. The results suggest that in certain cases, DBS is a potentially effective treatment option for affected patients. However, the level of evidence is low, since the data is primarily based on retrospective studies with small number of cases. Moreover, the definitions of postoperative outcomes are heterogenous between the existing studies, which impedes the comparison of the studies with one another. Controlled, prospective trials with statistical reliable numbers of patients are missing to give a clear recommendation whether DBS is a sufficient treatment of PD-associated postural abnormalities.

## Data Availability

All data generated or analyzed during this study are included in this article. Further inquiries can be directed to the corresponding author.
